# Rapid deployment of a mobile biosafety level-3 laboratory in Sierra Leone during the 2014 Ebola virus epidemic

**DOI:** 10.1371/journal.pntd.0005622

**Published:** 2017-05-15

**Authors:** Yi Zhang, Yan Gong, Chengyu Wang, Wensen Liu, Zhongyi Wang, Zhiping Xia, Zhaoyang Bu, Huijun Lu, Yang Sun, Xiaoguang Zhang, Yuxi Cao, Fan Yang, Haoxiang Su, Yi Hu, Yongqiang Deng, Bo Zhou, Zongzheng Zhao, Yingying Fu, David Kargbo, Foday Dafae, Brima Kargbo, Alex Kanu, Linna Liu, Jun Qian, Zhendong Guo

**Affiliations:** 1 Key Laboratory of Jilin Province for Zoonosis Prevention and Control, Changchun Veterinary Research Institute, Chinese Academy of Agriculture Sciences, Changchun, Jilin, China; 2 School of Materials Science & Engineering, Beijing Institute of Fashion Technology, Beijing, China; 3 National Institute for Viral Disease Control and Prevention, Chinese Center for Disease Control and Prevention, Beijing, China; 4 Institute of Pathogen Biology, Chinese Academy of Medical Sciences & Peking Union Medical College, Beijing, China; 5 State Key Laboratory of Pathogen and Biosecurity, Beijing Institute of Microbiology and Epidemiology, Beijing, China; 6 Sierra Leone Ministry of Health and Sanitation, Freetown, Sierra Leone; 7 Sierra Leone-China Friendship Hospital, Freetown, Sierra Leone; School of Veterinary Medicine University of California Davis, UNITED STATES

## Abstract

**Background:**

Ebola virus emerged in West Africa in December 2013. The high population mobility and poor public health infrastructure in this region led to the development of the largest Ebola virus disease (EVD) outbreak to date.

**Methodology/Principal findings:**

On September 26, 2014, China dispatched a Mobile Biosafety Level-3 Laboratory (MBSL-3 Lab) and a well-trained diagnostic team to Sierra Leone to assist in EVD diagnosis using quantitative real-time PCR, which allowed the diagnosis of suspected EVD cases in less than 4 hours from the time of sample receiving. This laboratory was composed of three container vehicles equipped with advanced ventilation system, communication system, electricity and gas supply system. We strictly applied multiple safety precautions to reduce exposure risks. Personnel, materials, water and air flow management were the key elements of the biosafety measures in the MBSL-3 Lab. Air samples were regularly collected from the MBSL-3 Lab, but no evidence of Ebola virus infectious aerosols was detected. Potentially contaminated objects were also tested by collecting swabs. On one occasion, a pipette tested positive for EVD. A total of 1,635 suspected EVD cases (824 positive [50.4%]) were tested from September 28 to November 11, 2014, and no member of the diagnostic team was infected with Ebola virus or other pathogens, including Lassa fever. The specimens tested included blood (69.2%) and oral swabs (30.8%) with positivity rates of 54.2% and 41.9%, respectively. The China mobile laboratory was thus instrumental in the EVD outbreak response by providing timely and reliable diagnostics.

**Conclusions/Significance:**

The MBSL-3 Lab significantly contributed to establishing a suitable laboratory response capacity during the emergence of EVD in Sierra Leone.

## Author summary

A Mobile Biosafety Level-3 Laboratory (MBSL-3 Lab) and a well-trained diagnostic team were dispatched to Sierra Leone to assist in Ebola virus disease (EVD) diagnosis when the largest outbreak of EVD to date emerged in West Africa in 2014. This setup allowed for the diagnosis of suspected EVD cases in less than 4 hours from the time of sample receiving. The laboratory was composed of three container vehicles and was equipped with advanced ventilation system, communication system, electricity and gas supply system. Multiple safety precautions were strictly applied to reduce exposure risks. A total of 1,635 suspected EVD cases were evaluated from September 28 to November 11, 2014, and none of the staff members was infected with Ebola virus or other pathogens. The China mobile laboratory was thus instrumental in the EVD outbreak response by providing timely and accurate diagnostics. Therefore, the MBSL-3 Lab played a significant role in establishing a suitable laboratory response capacity during the emergence of EVD in Sierra Leone.

## Introduction

Ebola virus belongs to the Filoviridae family of enveloped viruses and contains a non-segmented negative-strand RNA genome [1,2]. Infection in humans can cause Ebola hemorrhagic fever, with exceptionally high case-fatality rates of more than 50% [3,4]. The incubation period of Ebola virus disease (EVD) is 2 to 21 days [5]. The clinical signs and symptoms are extremely similar to those of the Marburg virus and include fever, body aches, vomiting, diarrhea, rash and, in some cases, both internal and external bleeding [5]. Patients usually die of multiple-organ failure or hypovolemic shock. No licensed therapeutic or prophylactic treatments are currently available.

The largest outbreak of EVD has been ongoing in West Africa since December 2013. As of April 15, 2015, 25,826 cases (10,704 deaths [41.4%]) had been reported by the World Health Organization (WHO) [6]. Although direct contact is the main route of transmission [7–10], EVD is still easily contagious, and healthcare workers have constituted a considerable proportion of all cases. In particular, by April 11, 2015, 864 healthcare workers (503 deaths [58.2%]) had been infected [6].

Ebola virus is classified as a biosafety level-4 agent. Clinical specimen inactivation should be performed in a biosafety level-3 laboratory, and subsequent to this step, routine testing can be performed in a biosafety level-2 laboratory. However, at the time of the outbreak, West Africa had few high-level biosafety facilities, so scientists had to work under difficult and dangerous conditions associated with potential exposure risks [11]. It would take a fairly long time, a large staff and many resources to construct a new fixed biosafety facility, thus delaying prevention and control of the epidemic. Therefore, a mobile unit [12,13] with both biosafety and flexibility was urgently needed to manage epidemics and emergent public health incidents such as the EVD outbreak.

In September 2014, China responded to the appeal made by the United Nations and WHO and offered assistance to the government of Sierra Leone. A truck-based mobile biosafety level-3 laboratory (MBSL-3 Lab) and a well-trained diagnostic team were then dispatched and deployed to the Sierra Leone-China Friendship Hospital, in one of the hardest-hit areas, near Freetown, to assist in EVD diagnosis. The team members and aid supplies arrived on September 17, 2014. It took approximately one week to rebuild part of the hospital into multiple functional regions to meet the specimen testing requirements, including a specimen-receiving region, a supply-storage region, a waste-incineration region, a nucleic-acid-detection region, and a staff-rest area, among others. The MBSL-3 Lab was transported by an airlift jet aircraft (Antonov An-124 Ruslan, Russia) from Beijing Capital International Airport on September 24, 2014, at 03:00 (Beijing time) to Freetown International Airport on September 25, 2014, at 14:00 (Freetown time), with a flight duration of 43 h. It took another three and a half hours to drive the MBSL-3 Lab to the Sierra Leone-China Friendship Hospital. With strict training and standard operating procedures (SOPs), clinical specimen testing began within 60 h after the arrival of the MBSL-3 Lab, enabling the diagnosis of suspected EVD cases in less than 4 hours from the time of sample receiving. In total, 1,635 suspected EVD cases (824 positive [50.4%]) were tested from September 28 to November 11, 2014, and none of the staff members was infected with Ebola virus or other pathogens. Here, we provide a brief overview of the MBSL-3 Lab and the biosafety precautions applied to manage the EVD outbreak.

## Methods

### Ethical statement

This Ebola outbreak response was a humanitarian aid mission. The SOPs used were approved by the WHO and the Sierra Leone Ministry of Health and Sanitation (MoHS). The diagnostic results were released immediately after the specimen analyses were completed.

### Specimen collection

Specimens were delivered to our worksite daily from two sources: the emergency operations center jointly established by the Sierra Leone MoHS and the China medical aid team who accompanied us and was also deployed to the Sierra Leone-China Friendship Hospital.

When picking up the specimens, the staff wore one layer of personal protective equipment (PPE), including a protective suit (Lakeland INC or DuPont, USA), an N95 mask (3M, USA), an anti-impact goggle (3M, USA), two pairs of latex gloves with the inner pair taped to the protective suit and a pair of dedicated shoes and waterproof shoe covers (S1 Fig). The surface of the specimen bucket and the packing bag were disinfected by spraying with 0.25% chlorine-containing disinfectants.

### Specimen inactivation and RNA extraction

The staff extracted RNA in the BSL-3 Lab wearing two layers of PPE. The inner PPE included a protective suit, an N95 mask, a pair of inner gloves and a pair of dedicated shoes and waterproof shoe covers (S1 Fig). The external PPE included a HEPA filter-equipped powered air purifying respirator (3M, USA), a disposable sterilized surgical gown, a pair of external gloves and waterproof shoe covers (S1 Fig). The specimen bucket was opened within the biosafety cabinet. As Buffer AVL in the QIAamp Viral RNA Mini Kit (Qiagen, Germantown, MD, USA) was insufficient to inactivate samples [14], a combination of physical and chemical inactivation was performed to enhance the inactivation efficiency. The specimens were first inactivated by incubation in a water bath at 62°C for 1h before opening the tube cap to pipette the samples and were then further inactivated by the addition of Buffer AVL to the samples.

RNA was extracted using the QIAamp Viral RNA Mini Kit (Qiagen, Germantown, MD, USA) according to the manufacturer’s protocol. All waste was first chemically inactivated (with 0.25% chlorine-containing disinfectant), then sterilized using a double-leaf autoclave and finally incinerated.

### Q-RT-PCR diagnostic assays

Quantitative real-time PCR (Q-RT-PCR) assays were performed using a set of published primers and probes [15], targeting regions of the glycoprotein gene (F: 5’-TGGGCTGAAAAYTGCTACAATC-3’; R: 5’-CTTTGTGMACATASCGGCAC-3’; Probe: FAM-5′-CTACCAGCAGCGCCAGACGG-3′-TAMRA). RNA was amplified using the One Step PrimeScript RT-PCR Kit (TaKaRa, Japan), and 40-cycle Q-RT-PCR assays were run on the LightCycle 96 System (Roche, Switzerland). Melt curve analysis was performed to confirm the identity of the amplification products. The specimens were considered positive if there was an apparent logarithmic phase in the amplification curve, with melting point confirmed amplification products and the Ct value≤36 (Ct value<26, intense positive; 26≤Ct value≤ 36, weak positive). In contrast, the specimens were considered negative if there was no apparent logarithmic phase, with the Ct value undetermined, and they were considered suspect when 36<Ct value≤40.

### Specimen storage

The MBSL-3 Lab was equipped with a -20°C freezer and a -80°C freezer, and there was another -80°C freezer outside the MBSL-3 Lab. As a result, we could store a total of 1500–2000 specimens. For short-term storage, namely, within 1 day, we stored the specimens at -20°C. For long-term storage, we stored the specimens at -80°C. The specimens were well packed and surface disinfected with 0.25% chlorine-containing disinfectant before storage. The Sierra Leone-China Friendship Hospital was guarded by the military guard of Sierra Leone, and the freezers were well locked.

### Testing report release

Every patient was assigned a unique Outbreak Case ID by the emergency operations center jointly established by the MoHS. Each time a sample was collected, the patient was asked to complete a “VIRAL HEMORRHAGIC FEVER CASE INVESTIGATION FORM”. The sample tube and the investigation form were marked with the Outbreak Case ID and patient name and were then delivered to us. Therefore, the Outbreak Case ID provided a unique number for tracking the patient, the specimen and the test result. The information in our testing report included the Outbreak Case ID, the Ct value yielded by Q-RT-PCR and the confirmed result (Yes/No/Suspect).

According to an agreement with the MoHS, we usually did not contact hospitals directly. Instead, we submitted the testing report to the WHO and the MoHS, which was in charge of delivering the results to hospitals. In particular, the China medical aid team who came with us and was also deployed to the Sierra Leone-China Friendship Hospital could get testing results from us directly.

### Biosafety risk assessment

#### Air sample collection

To assess the aerosol exposure risk of the working environment, we used the SASS 4100 air sampler (3,500L/min) and SASS 3010 filter extractor (Research International, Inc., USA) to collect air samples from the MBSL-3 Lab approximately every 15 days (for a total of 3 times).

The virus was then concentrated by polyethylene glycol (PEG) precipitation. In brief, the pH of the virus-containing supernatant was adjusted to 7.2–7.5, after which PEG (MW 6,000) was added to a final concentration of 8%. The samples were stirred at 4°C for 2 hours and then centrifuged at 10,000 g for 2 hours. Finally, the pellet was resuspended in 400 μl of RNase-free ddH_2_O for virus RNA preparation.

#### Swab collection

To assess the contamination risk during the experimental operations, we collected swabs from the surfaces of potentially contaminated objects, one or two sites per workday, including experimental gloves, pipettes, workbenches, doorknobs, centrifuges and specimen buckets. The swabs were placed in phosphate-buffered saline (PBS)-containing penicillin (100U/ml) and streptomycin (100μg/ml).

## Results

### Staff composition and worksite layout

**Fig 1:**
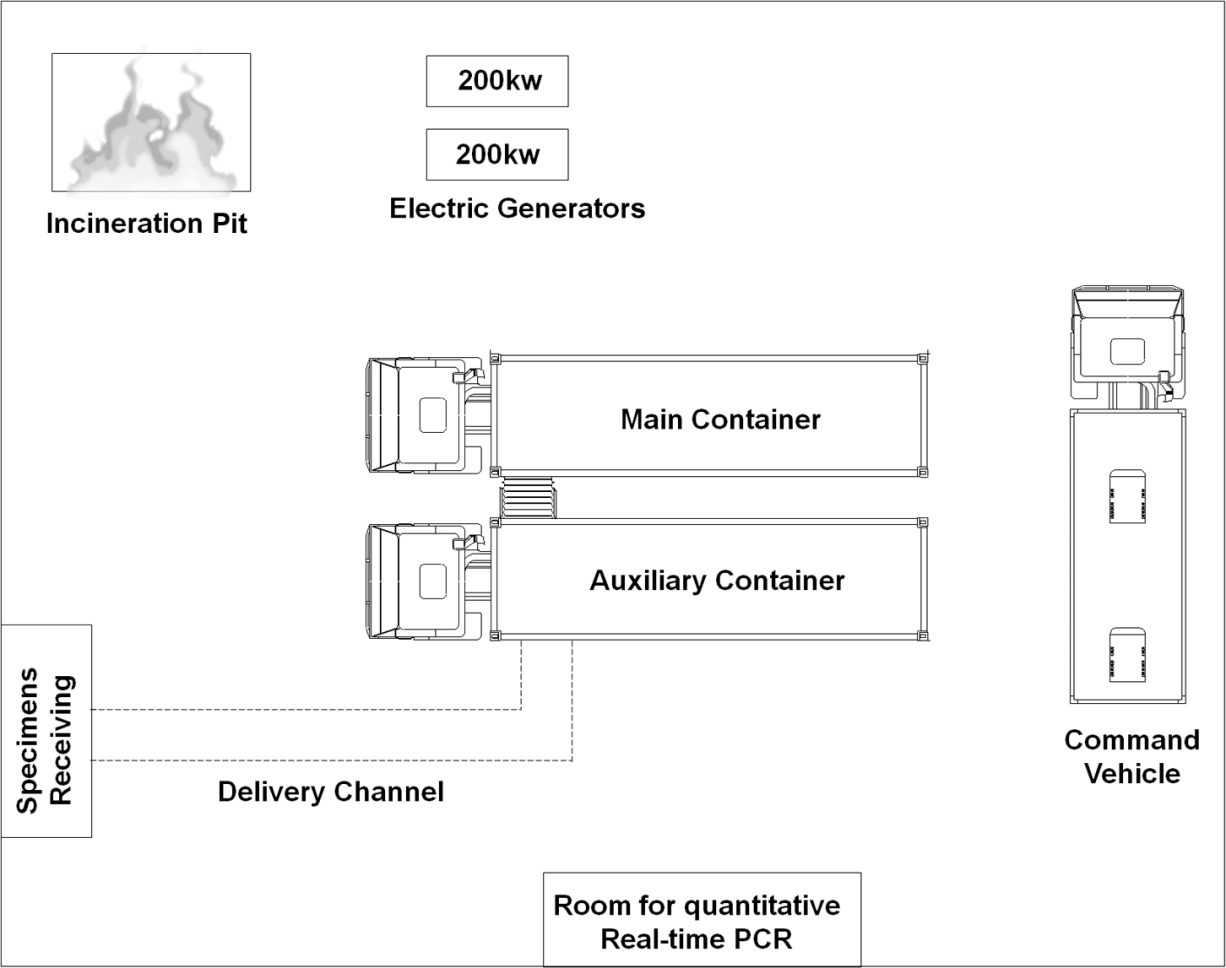
Worksite layout for the China mobile laboratory diagnostic team.

The China MBSL-3 Lab arrived in Sierra Leone on September 25, 2014, and specimen tests were carried out within 60 h of its arrival. The worksite layout was shown in Fig 1. After receiving specimens, scientists sent them to the MBSL-3 Lab, where RNA was extracted. One room in the hospital was rebuilt and used for subsequent Q-RT-PCR analysis. The MBSL-3 Lab was powered by alternate use of 200kW diesel generators. Lab and household trash was incinerated away from the lab or structures in a pit. There were surveillance cameras all around the worksite and inside every experimental room, and scientists could watch real-time surveillance video and communicate with the experimenters in the laboratory.

An overview of the composition of the China mobile laboratory diagnostic team and the team members’ tasks was shown in Table 1. One scientist was in charge of contacting the MoHS to coordinate issues such as sending specimens and releasing analysis results. In addition, eight scientists engaged in virus detection. Technical support personnel were in charge of the operation of the MBSL-3 Lab, including overseeing the water and electricity supply, maintenance and repair of equipment, sterilization and incineration of lab trash as well as watching and recording the daily experimental process. Two medical doctors monitored the health conditions of every staff member.

**Table 1:** Overview of the China mobile laboratory diagnostic team’s composition and their tasks.

Task		Staff member(s)
External contact		1×Scientist
Virus diagnosis	• Sample receiving	8×Scientists
	• RNA extraction	
	• Q-RT-PCR analysis	
	• Info check and data control	
Technical support		3×Scientists, 1×Assistant
Health care		2×Medical doctors

### Performance of the MBSL-3 Lab

**Fig 2:**
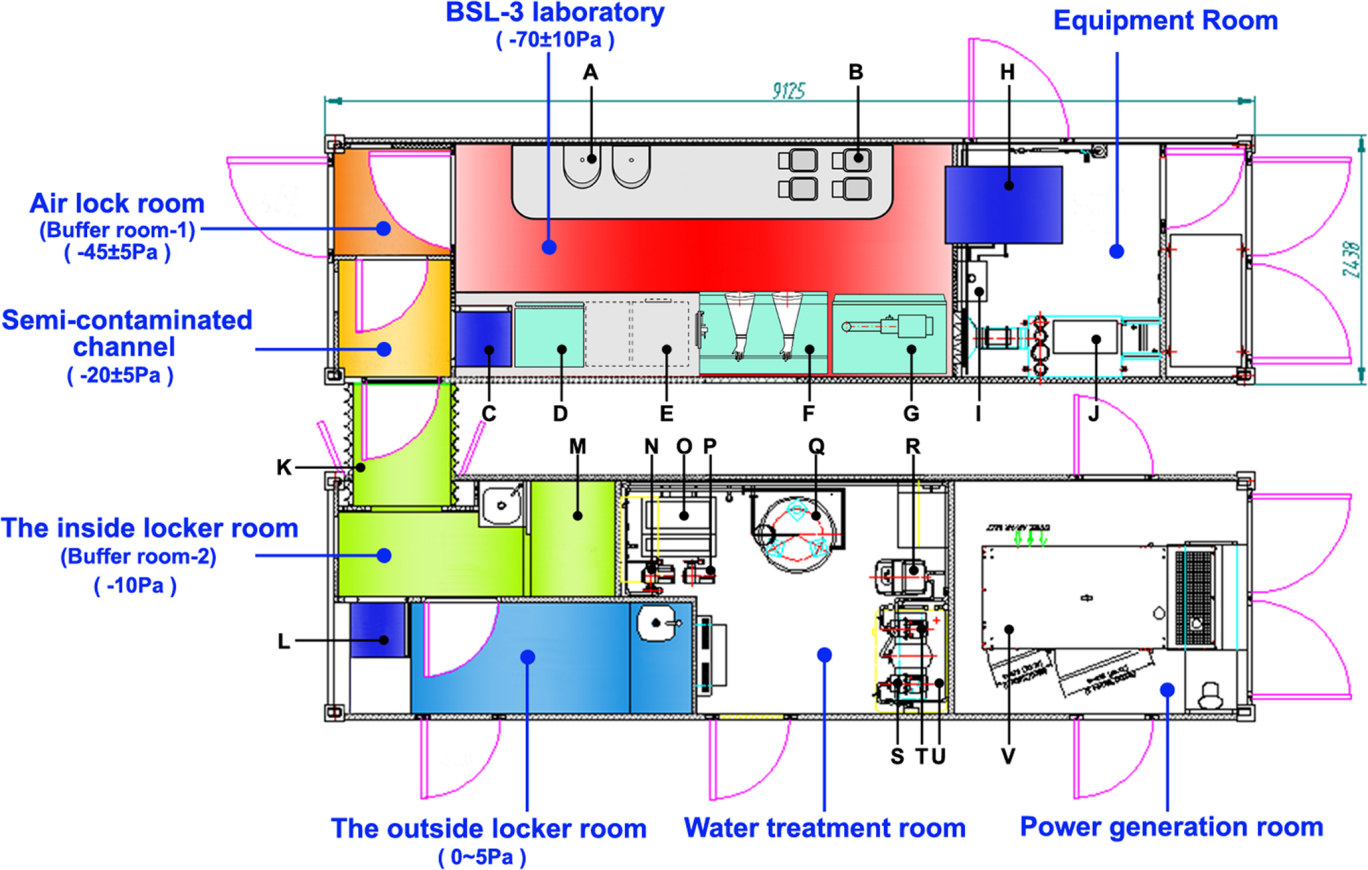
Layout of the mobile biosafety level-3 laboratory. The main and auxiliary containers were connected by an airtight soft connection and together formed a complete biosafety level-3 (BSL-3) lab. The instruments represented by letters were listed in S1 Table.

The MBSL-3 Lab was composed of three container vehicles. The container encompassing the BSL-3 laboratory was called the main container (L×W×H: 9125×2438×2896mm); the second container, of the same size, was used for personnel cleaning and technology support and was called the auxiliary container; and the third container was the command container (L×W×H: 6300×2460×2100mm). As shown in Fig 2, the main and auxiliary containers were connected by an airtight soft connection and together formed a complete BSL-3 Lab. From the entrance to the inside, in order, there was the outside locker room (0-5Pa), the inside locker room (Buffer room-2, -10Pa), the semi-contaminated channel (-20±5Pa), the air lock room (Buffer room-1, -45±5Pa) and the BSL-3 laboratory (-70±10Pa). The doors were interlocking.

The checklist for the different workplaces and instruments in the MBSL-3 Lab was listed in S1 Table. The MBSL-3 Lab provided triple protection for humans, specimens and the environment. The main performance of the MBSL-3 Lab was detailed as follows.

#### • Power system

The power supply for the MBSL-3 Lab could be provided by two means: the diesel generator and the mains supply, which could switch automatically to ensure a timely and reliable supply. In the case of sudden power loss, the Uninterruptible Power Supply (UPS) could power the ventilation, illumination, auto-control system and experimental equipments for at least 45 min. Due to the lack of a stable mains supply in Sierra Leone, we employed two diesel-generating sets (200kw) for this mission.

#### • Ventilation system

A cascade of low pressure with steps of 0 Pa, -10 Pa, -20 Pa, -45 Pa and -70 Pa was realized with a ventilation system using 100% fresh filtrated air, allowing an air renewal rate of up to 20 times per hour. The air supply was purified by three-grade-filtration, namely, a primary efficiency filter (G4, plate-type), a medium efficiency filter (F8, pocket-type) and a high efficiency filter (H14, HEPA). The exhaust was purified by two-grade-filtration, namely, a high efficiency filter and a BAG-IN/BAG-OUT Filter Housing. The static cleanliness of the laboratory was up to International Standard Organization (ISO) Class 8 [16]. The MBSL-3 Lab was also equipped with an air conditioning system, which was a big selling point for laboratory personnel working in tropical climates. The temperature regulation range was from 16°C to 30°C.

#### • Waterway system

The water storage tank in the auxiliary container could store 2 tons of water. The water supply for the autoclave, vapor generator and air conditioning humidifier was softened using a water softener. Wastewater from hand washing and the shower was stored in the wastewater tank directly under the shower cubicle and could be pumped into the sewage treatment tank by the vacuum pump for sterilization. The sewage treatment tank had two sterilization methods: high-temperature vapor and chemical sanitizer.

#### • Gas supply system

Compressed air generated by the air compressor served the double-leaf autoclave (STERIS, Amsco Century) and expanded-metal door. There were two 8-Liter CO_2_ gas bombs that provided continuous CO_2_ to the incubator.

#### • Communication system

Instruments, logic controllers and industrial control computers together formed a data acquisition and monitoring system. The status and data of the MBSL-3 Lab, including the pressure, temperature, humidity, resistance of filter, working state of the blower, biosafety cabinet and glove box, as well as fault inquiries, could be transmitted to the command container though signal lines and was monitored using “King View” industry control software. In addition, our MBSL-3 Lab was equipped with an internal communications network and a video surveillance system. Telephone calls, data transfer and faxes between the BSL-3 laboratory and the command container could be completed via a local area network.

#### • Modes of transportation

The MBSL-3 Lab can be transported by air, land and sea. It can be operated on trucks, or can be dismounted to be operated on the ground. In the latter case, hoisting with a crane and self-lifting by four elevating motors are two methods that can be used for dismounting to the ground (S2 Fig). When the MBSL-3 Lab is needed in a remote setting that cannot be reached by land transportation due to impassable roads, it could be transported by hoisting with a helicopter, similar to how it is lifted by a crane, as shown in S2C Fig.

**Fig 3:**
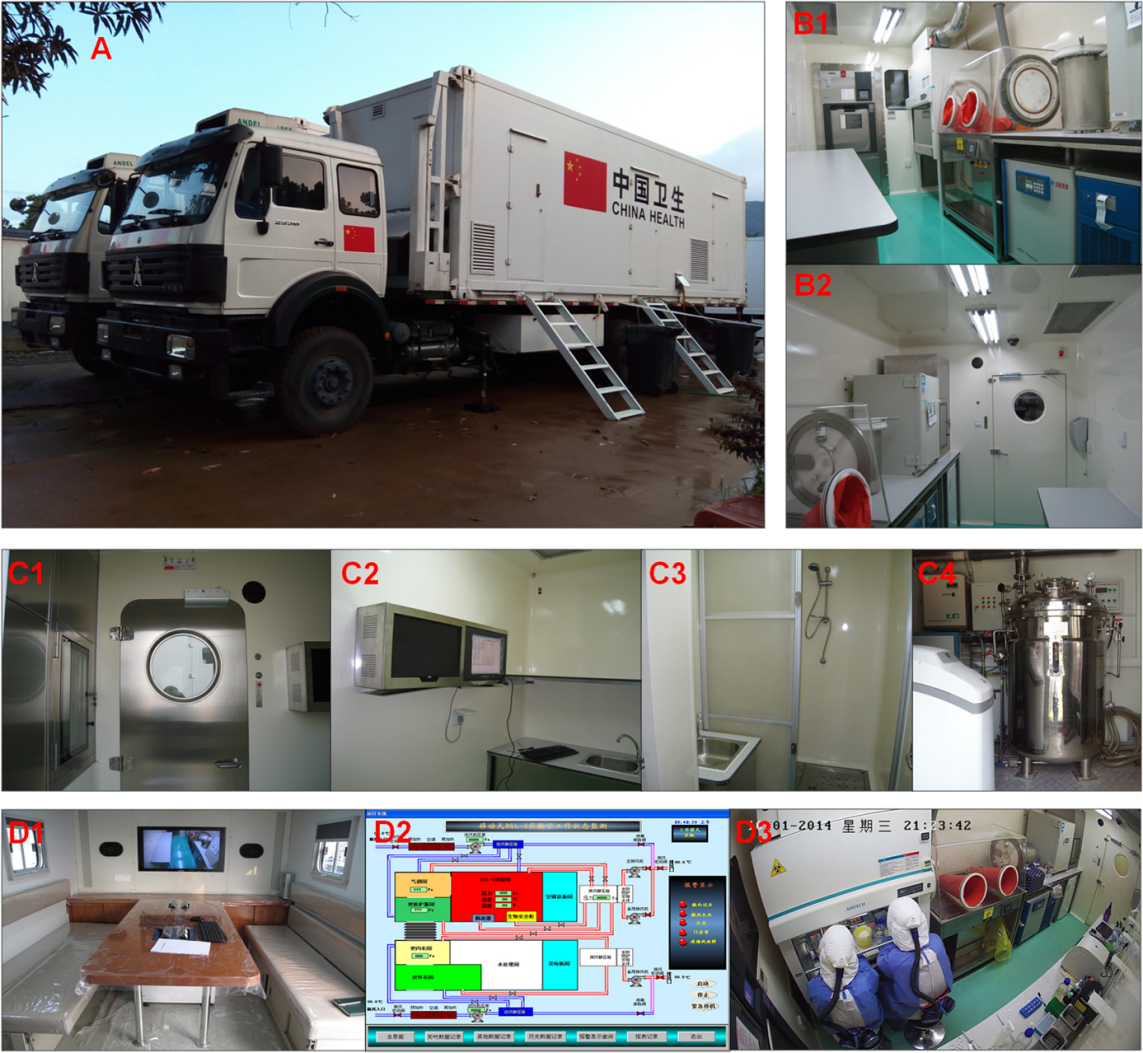
Mobile biosafety level-3 laboratory at its mission in Sierra Leone. (A) Exterior of the mobile biosafety level-3 laboratory. (B) View to the biosafety level-3 laboratory. Two different perspectives (B1 and B2) were shown. (C) View to the auxiliary container. C1) Pass box (left) and expanded-metal door (middle). C2) Monitoring unit and table for experimental preparation. C3) Shower cubicle. C4) Waste treatment room. (D) View to the command container. D1) Room for meeting or for watching monitoring videos. D2) “King View” industry control software. D3) Real-time surveillance video.

The MBSL-3 Lab at its mission in Sierra Leone was shown in Fig 3.

### Biosafety risk management

**Fig 4:**
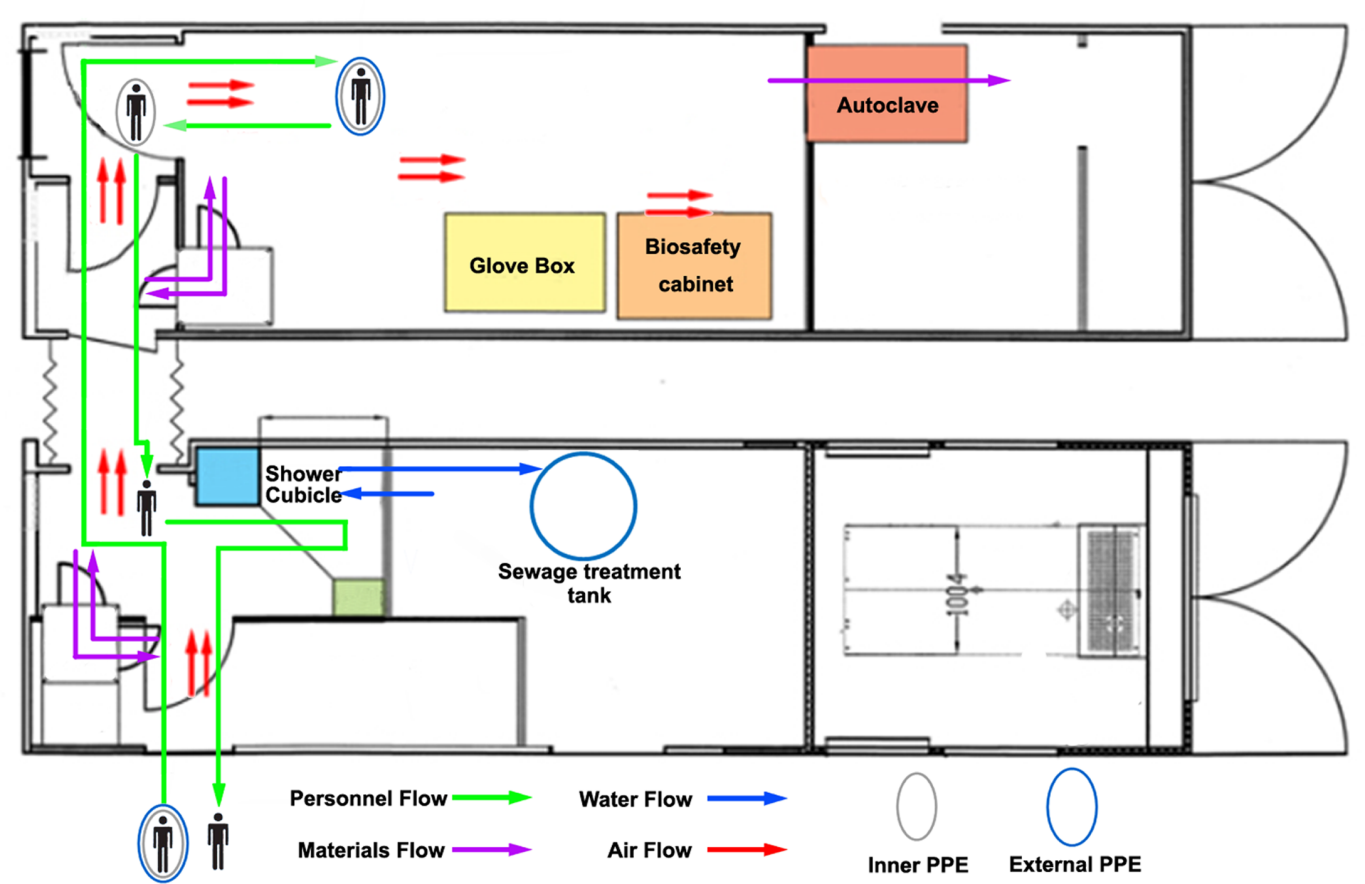
Schematic diagram of the “Four Flows”. Personnel, materials, water and air flow management were the key elements of biosafety measures in the MBSL-3 Lab.

“Four Flows” management were the key elements of biosafety measures in the MBSL-3 Lab (Fig 4).

#### Personnel flow

No more than 2 experimenters were allowed inside the BSL-3 Lab to avoid influencing the air-flow. After completing an experiment, a step-reducing contamination process was carried out for the experimenters to exit the BSL-3 Lab. More specifically, the experimenters disinfected the surface of their PPE with chlorine-containing disinfectants in the BSL-3 lab and waited in Buffer room-1 for 10 min to ensure adequate disinfection, after which they doffed their external PPE. Then, in Buffer room-2, they removed the inner PPE and took a shower.

#### Materials flow

Lab supplies were brought in sterilely though pass boxes to avoid contaminating experimental objects. Lab trash was chemically inactivated and removed through the double-leaf autoclave after sterilization. The sterilization effect was monitored using autoclave indicator tape (3M, USA). RNA samples were well packed and then surface disinfected and taken out through pass boxes.

#### Water flow

Water was supplied after purification, and one-way flow was guaranteed by pressure pumps and check valves. Waste water was discharged after steam sterilization and chemical disinfection.

#### Air flow

Intake and exhaust air was HEPA filtered. The cascade of low pressure formed directional airflow from the outside to the inside. The diagonal ventilation of up-supply and down-discharge ensured uniform airflow and no dead corners. The air supply was controlled by a constant air volume (CAV) system, and air exhaust was controlled by a variable air volume (VAV) system, which reduced the possibility of instantaneous positive pressure.

### Risk assessment of the working environment and experimental operation

To assess the aerosol exposure risk when working in or around the MBSL-3 Lab, air samples were collected from the BSL-3 lab, locker rooms, water treatment room, equipment room, exhaust outlet and command container and were concentrated for EVD detection every 15 days (S1 Fig). Fortunately, all results were negative.

We also collected swabs from the surfaces of potentially contaminated objects to determine whether there was an existing exposure risk (S2 Table). On one occasion, the pipette used to pipette samples from the blood-collection tubes tested positive for EVD, with a Ct value of 27.75.

### Other protective measures

#### Disinfection

Approximately every ten days, on October 5, 15, and 24 and November 5, 2014, we maintained the MBSL-3 Lab and did not perform Q-RT-PCR assays. On these four days, a thorough disinfection of the MBSL-3 Lab was performed to maintain a safe working environment. The BSL-3 Lab was disinfected by fumigation with peroxide hydrogen and the *Geobacillus stearothermophilus* spores were used as biological indicator (Mesa Labs, USA) to monitor the disinfection effect. The buffer rooms were disinfected by spraying with peracetic acid.

#### Health care

Each staff member had his or her own personal space in which to rest between shifts. The work arrangement also ensured that the staff had sufficient rest time and that their health conditions were carefully tended to.

#### Disease prevention and control

Each staff member carried an alcohol sprayer to disinfect potentially contaminated sites at any moment. To keep the staff members healthy and free from other infectious diseases, such as malaria, yellow fever, Lassa fever and typhoid fever, the worksite was sanitized using the DEMAND capsule suspension to control mosquitoes, and mousetraps were used to control rodents (S1 Fig).

### Workflow for laboratory testing and test results

**Fig 5:**
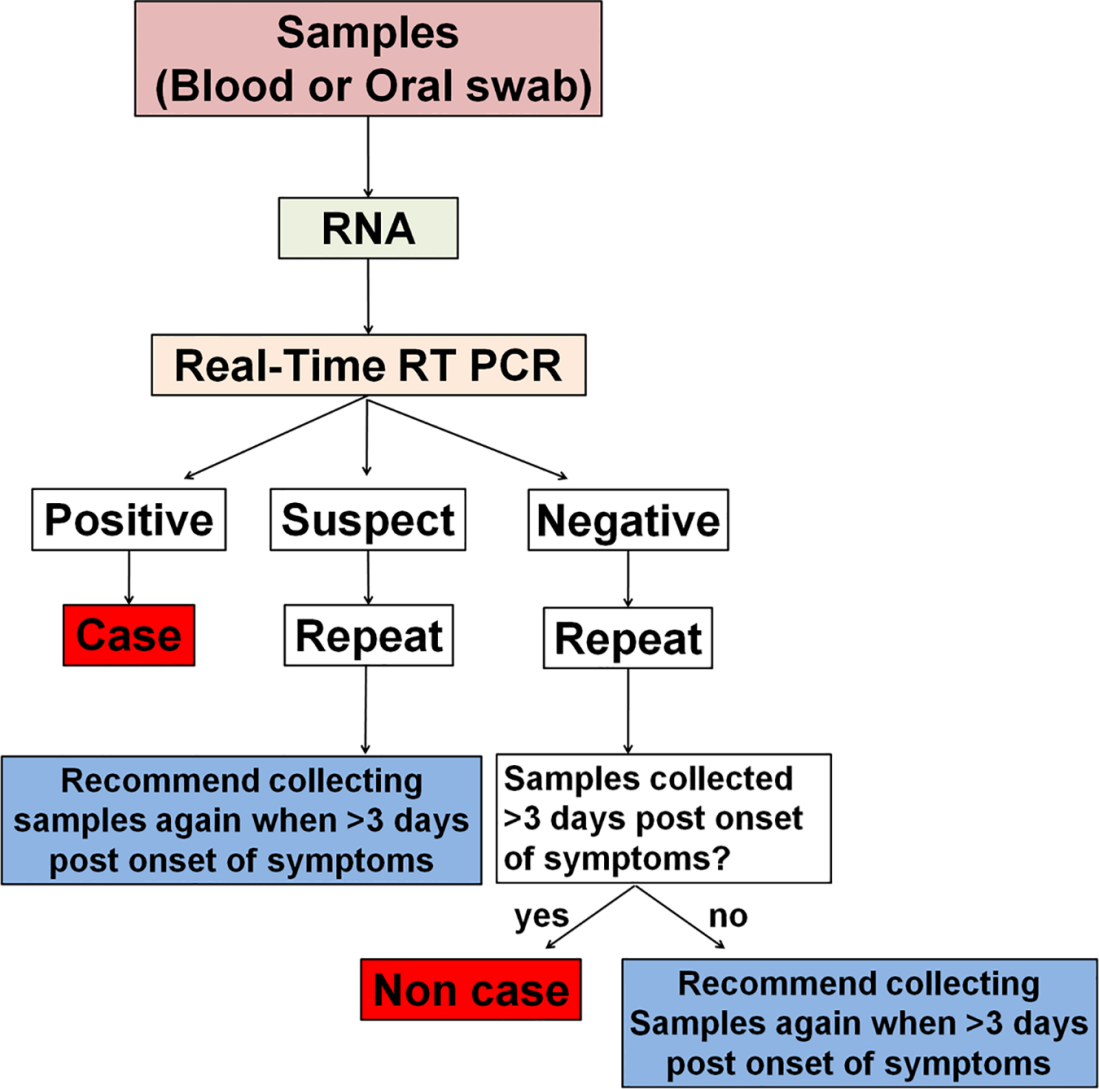
Diagnostic algorithm for laboratory testing.

The diagnostic algorithm for laboratory testing and the rationale for positive/negative/suspect test results were presented in Fig 5. We repeated the testing of the suspect and negative cases and strongly recommended collecting specimens again if collection was performed <3 days post onset of symptoms. We found no evidence of RNA contamination during the entire operation. We added positive and negative controls to every experiment, and all controls produced the expected results.

Overall, 1,635 suspected EVD specimens were tested from September 28 to November 11, 2014, primarily blood/serum samples (69.2%) and oral swabs (30.8%). The sample sources and test results were presented in Table 2. In total, 824 cases (50.4%) were identified as positive, and the positive rate of the swab samples (41.9%) was slightly lower than that of the blood samples (54.2%).

**Table 2:** Samples and test results from September 28 to November 11, 2014.

Samples	Total samples tested	Positive/Negative/Suspect	Positive rate
Blood samples	1,131	613/446/72	54.2%
Swab samples	504	211/280/13	41.9%
Total	1,635	824/726/85	50.4%

The positive rate was defined as the number of positive cases divided by the total number of samples.

## Discussion

The number of various paroxysmal public health events has been growing, and most have occurred in poverty-stricken areas. However, the resources for medical treatment, outbreak management and laboratory research are concentrated in developed regions, and substantial expenditure would be required to build new medical systems in these areas. Because epidemic situations are always urgent, scientists thus work under inadequate conditions and face exposure risks. Therefore, rapid, safe and flexible outbreak response capacity is urgently needed [17]. A mobile laboratory unit can easily be promptly deployed when needed and can provide a safe working environment, which will be a vital part of the outbreak response to emerging public health events or bioterrorism acts and will make great contributions to lessening and controlling epidemics. Several mobile units have previously been used in natural disaster scenarios [18,19], in health surveys [20,21], during the outbreak of severe infectious diseases [22–24] and in military campaigns [25].

Our MBSL-3 Lab meets the requirements of on-site collection, isolation, cultivation and detection of emergent infectious pathogens. This laboratory also protects humans as well as the environment and specimens, and it was designed to be functional in a field setting, even without logistical support. The major challenges in a remote location may be power supply and water supply, but there are ways to overcome them. There was an 80kVA (≈70kW) diesel generating set in the auxiliary container of the MBSL-3 Lab. Full fuel in the oil box can power the MBSL-3 Lab in continuous operation for 24h. We can bring as much fuel with us as possible using oil tanks, and wherever the MBSL-3 Lab can arrive, a refueling truck could also arrive. The MBSL-3 Lab is also equipped with a water storage tank and a water softener, and water can be re-supplied with water from a well or clear stream. If the experimenters could do not take a shower in the MBSL-3 Lab, the water requirement is not large, approximately 200L per day. In addition, the MBSL-3 Lab is equipped with a leveling system, but it still needs a 20m×8m level ground. This was the first time that we executed a mission in Africa. In total, 1,635 specimens were tested from September 28 to November 11, 2014, accounting for more than one quarter of the nation’s specimen volume during the same period. In all, 824 (50.4%) specimens were EVD-positive, representing 33.3% of the total number of confirmed cases reported in Sierra Leone during the same period. The maximum number of specimens that we could reasonably process in one day is approximately 120–150.

We developed strict SOPs, adopted comprehensive protective measures and used comprehensive medical and logistical support systems to ensure safe and orderly performance of the virus diagnosis task. In particular, the “Four Flows” biosafety protocol was strictly followed. We monitored the exposure risk during clinical specimen testing. Air samples were collected from every workspace, and the test results were all negative, indicating that the working environment was relatively safe. The surfaces of potentially contaminated objects were also swabbed. On one occasion, the pipette used to pipette samples from blood-collection tubes tested positive. Given that a portion of the specimens contained only a small sample volume, the pipette had to be placed deep into the tubes and was easily contaminated by touching the inner wall. Therefore, it was suggested that the barrel of the pipette should be disinfected with disinfectant-containing gauze after pipetting each sample to avoid personnel infection and cross-contamination of samples.

The test results played an important role in the disposal of symptomatic individuals and might, in a sense, determine their fates. For positive cases, the patients would be properly isolated and treated without visiting family members, and traditional religious funerals for the dead were forbidden. For negative cases, the patients would be separated from the positive cases and kept in an observation ward for follow-up testing or discharge to relieve the limited wards. Hence, the accuracy of the test results was crucial. False-positive results might lead to the individual being infected by positive patients, whereas false-negative results might lead to the spread of EVD to families and even the community. Our diagnostic algorithm suggested a suspect conclusion when 36<Ct value≤40 and strongly recommended resampling and considering clinical information and epidemiological links. Q-RT-PCR is now a preferred method for pathogen diagnosis due to its rapid and sensitive features [26], but it is prone to contamination and may result in false-positive results. Therefore, we conducted every experiment in the biosafety cabinet. The cabinet and PCR room were exposed periodically to ultraviolet radiation to eliminate nucleic acid contamination. Additionally, PCR tubes were never opened. Every control included in the PCR assays produced the expected result, indicating high experimental accuracy. Moreover, the MoHS was in charge of retrospective look at the disease progresses of the patients, and to date, we have not received any feedback regarding a false diagnostic case from the MoHS.

We have shown that the positive rate of oral swabs was lower than that of blood samples. The technique and efficiency of swabbing might be one of the most important factors. Swab samples should be obtained by vigorous sampling to acquire sufficient biologic material for testing [27]. A quality-control PCR target (housekeeping gene target), such as Beta 2 Microglobulin (B2M), should be added for sample integrity assessment in the future.

Our MBSL-3 Lab continuously worked for six months and managed 4,867 specimens for EVD diagnostics. During that time, the China CDC established a fixed BSL-3 Lab near the Sierra Leone-China Friendship Hospital for long-term surveillance and to serve as the public health system for future outbreaks and epidemics. Currently, the EVD epidemic situation is under effective control, and our MBSL-3 Lab has been proven to be an important force for disease control and emergency disposal.

## Supporting information

S1 Fig Biosafety risk management. **(**A) Personal protective equipment (PPE) used when receiving specimens. (B) Inner PPE (B1) and external PPE (B2) used when extracting RNA. (C) Air samples were collected from every working room, including the biosafety level-3 laboratory (C1), equipment room (C2) and wastewater treatment room (C3). (D)The worksite (D1) and personal space in which to rest between shifts (D2) were completely sanitized using the DEMAND capsule suspension.
(TIF)

S2 Fig Working modes of the mobile biosafety level-3 laboratory. The mobile biosafety level-3 laboratory can be operated on trucks (A) or can be dismounted to be operated on the ground (B). Hoisting with a crane (C) and self-lifting by four elevating motors (D) are two methods that can be used for dismounting to the ground.
(TIF)

S1 Table Checklist for the workplaces and instruments of the mobile biosafety level-3 laboratory. (DOCX)

S2 Table Swab sampling sites among potentially contaminated objects and analysis results. (DOCX)
