# Supplementary figures and images for: Rapid deployment of a mobile biosafety level-3 laboratory in Sierra Leone during the 2014 Ebola virus epidemic
Source: PLoS Negl Trop Dis. 2017 May 15;11(5):e0005622. doi: 10.1371/journal.pntd.0005622 (PMC5444861; doi:10.1371/journal.pntd.0005622)

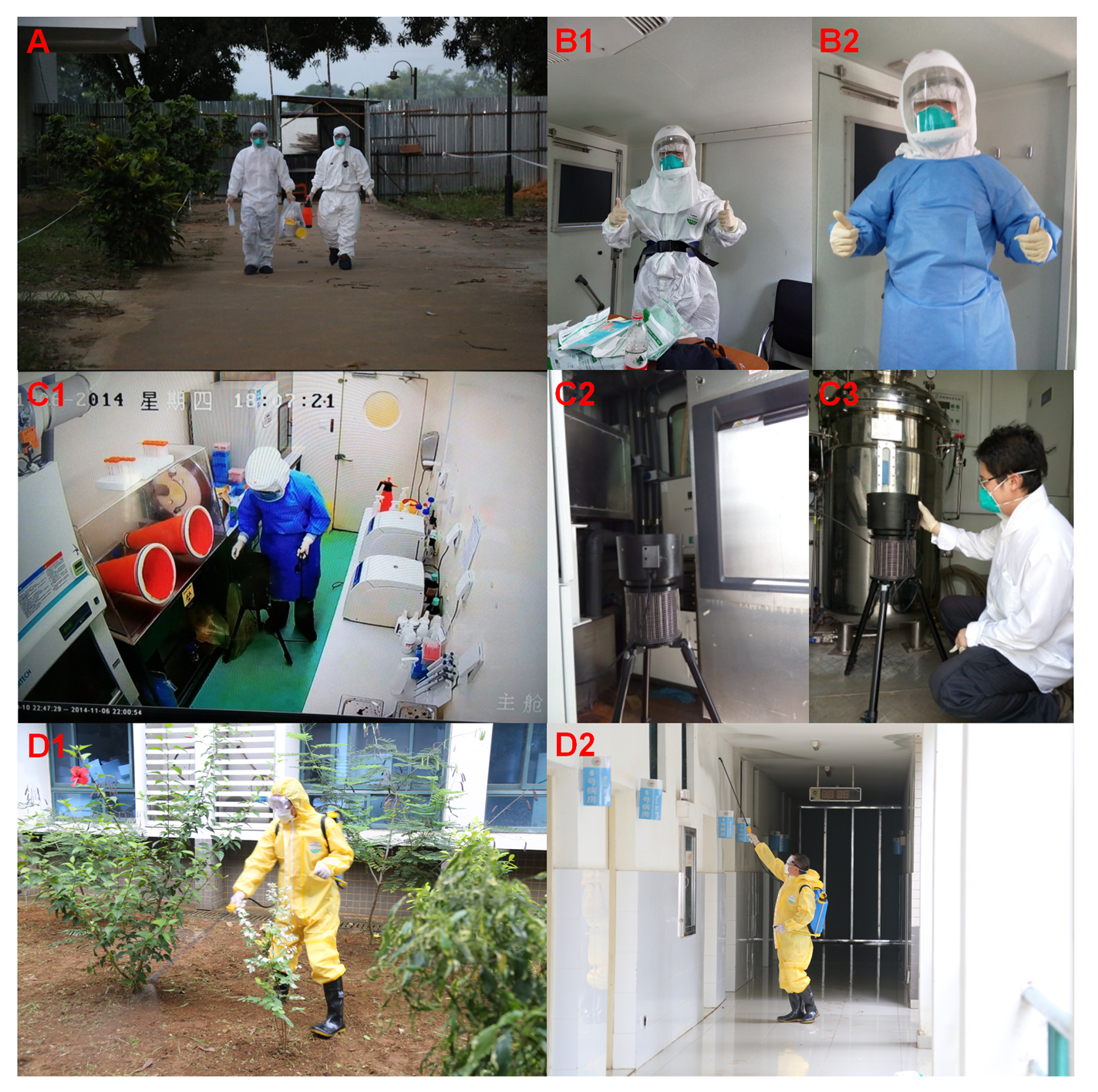

Supplement: S1 Fig — (A) Personal protective equipment (PPE) used when receiving specimens. (B) Inner PPE (B1) and external PPE (B2) used when extracting RNA. (C) Air samples were collected from every working room, including the biosafety level-3 laboratory (C1), equipment room (C2) and wastewater treatment room (C3). (D)The worksite (D1) and personal space in which to rest between shifts (D2) were completely sanitized using the DEMAND capsule suspension. (TIF) [file pntd.0005622.s001.tif]

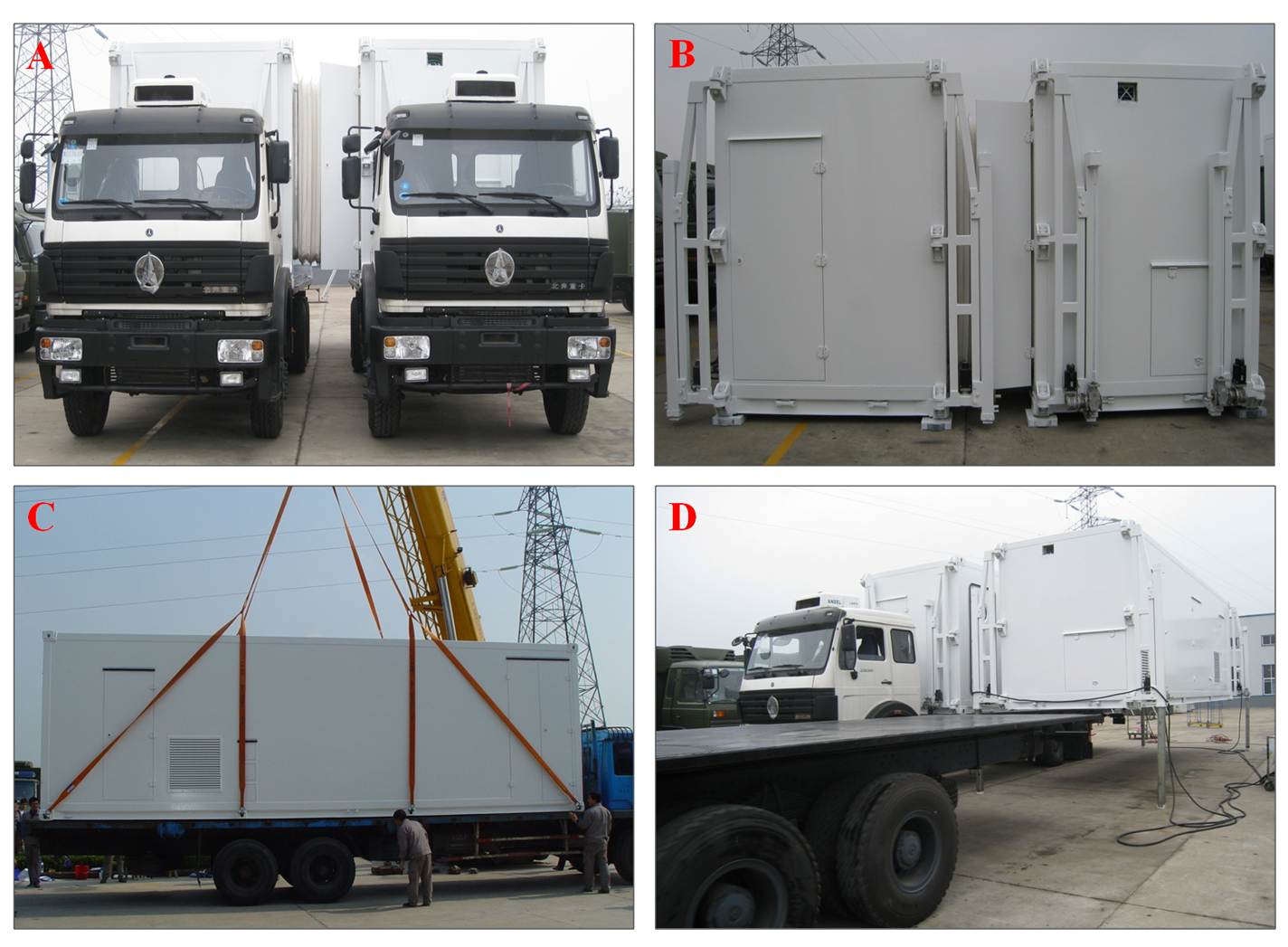

Supplement: S2 Fig — The mobile biosafety level-3 laboratory can be operated on trucks (A) or can be dismounted to be operated on the ground (B). Hoisting with a crane (C) and self-lifting by four elevating motors (D) are two methods that can be used for dismounting to the ground. (TIF) [file pntd.0005622.s002.tif]
