# Supplementary material for: Rapid deployment of a mobile biosafety level-3 laboratory in Sierra Leone during the 2014 Ebola virus epidemic
Source: PLoS Negl Trop Dis. 2017 May 15;11(5):e0005622. doi: 10.1371/journal.pntd.0005622 (PMC5444861; doi:10.1371/journal.pntd.0005622)
Supplement: S1 Table — (DOCX) [file pntd.0005622.s003.docx]

**S1 Table.** Checklist for the workplaces and instruments of the mobile biosafety level-3 laboratory

| Workplace | | Instrument | Letter in Fig 2 |
| --- | --- | --- | --- |
| Main container | Biosafety level-3 (BSL-3) laboratory | 2×Centrifuge | A |
|  |  | 4×Water bath | B |
|  |  | 1×Pass Box | C |
|  |  | 1×Biosafety cabinet (B2 type) | G |
|  |  | 1×Biosafety III Glove Box Isolator | F |
|  |  | 1×-80°C freezer, 1×-20°C freezer | E |
|  |  | 1×CO_2_ incubator | D |
|  |  | 1×Handsfree Disinfection | Not Shown |
|  |  | 2× Chairs | Not Shown |
|  | Equipment Room | 1×Double leaf autoclave | H |
|  |  | 1× UPS | I |
|  |  | 1× Ventilation and air conditioning system for BSL-3 lab | J |
| Auxiliary container | Buffer Room-2 | 1×Airtightly soft connection | K |
|  |  | 1×Pass Box | L |
|  |  | 1×Shower Cubicle | M |
|  | Water Treatment Room | 1×Water storage tank | U |
|  |  | 1×Self-priming pump | S |
|  |  | 1×Pressure pump | T |
|  |  | 1×Air compressor | P |
|  |  | 1×Vacuum pump | N |
|  |  | 1×Water heater | Not shown |
|  |  | 1×Water softener | R |
|  |  | 1×Steam generator | O |
|  |  | 1×Sewage treatment tank | Q |
|  |  | 1× Ventilation and air conditioning system for auxiliary container | Not shown |
|  | Power Generation Room | 1×Diesel generating set (80kVA) | V |
