# Supplementary material for: Rapid deployment of a mobile biosafety level-3 laboratory in Sierra Leone during the 2014 Ebola virus epidemic
Source: PLoS Negl Trop Dis. 2017 May 15;11(5):e0005622. doi: 10.1371/journal.pntd.0005622 (PMC5444861; doi:10.1371/journal.pntd.0005622)
Supplement: S2 Table — (DOCX) [file pntd.0005622.s004.docx]

**S2 Table**. Swab sampling sites among potentially contaminated objects and analysis results

| Sampling Site | Sampling frequency | Positive cases |
| --- | --- | --- |
| Gloves | 10 | 0 |
| Pipettes | 6 | 1 |
| Workbench | 9 | 0 |
| Doorknob | 4 | 0 |
| Centrifuge | 4 | 0 |
| Specimen bucket | 8 | 0 |
